# Effect of Regular Circus Physical Exercises on Lymphocytes in Overweight Children

**DOI:** 10.1371/journal.pone.0120262

**Published:** 2015-03-31

**Authors:** Cesar Miguel Momesso dos Santos, Fábio Takeo Sato, Maria Fernanda Cury-Boaventura, Silvia Helena Guirado-Rodrigues, Kim Guimaraes Caçula, Cristiane Cassoni Gonçalves Santos, Elaine Hatanaka, Heloisa Helena de Oliveira, Vinicius Coneglian Santos, Gilson Murata, Cristina Neves Borges-Silva, Sandro Massao Hirabara, Tania Cristina Pithon-Curi, Renata Gorjão

**Affiliations:** 1 Institute of Physical Activity Sciences and Sports, Post-Graduate Program in Human Movement Sciences, Cruzeiro do Sul University, São Paulo, Brazil; 2 Department of Physiology and Biophysics, Institute of Biomedical Sciences, University of São Paulo, São Paulo, Brazil; University of the Balearic Islands, SPAIN

## Abstract

Obesity associated with a sedentary lifestyle can lead to changes in the immune system balance resulting in the development of inflammatory diseases. The aim of this study was to compare lymphocyte activation mechanisms between overweight children practicing regular circus physical exercises with non-exercised children. The study comprised 60 pubescent children randomly divided into 4 groups: Overweight Children (OWC) (10.67 ± 0.22 years old), Overweight Exercised Children (OWE) (10.00 ± 0.41 years old), Eutrophic Children (EC) (11.00 ± 0.29 years old) and Eutrophic Exercised Children (EE) (10.60 ± 0.29 years old). OWE and EE groups practiced circus activities twice a week, for 4.3 ± 0.5 and 4.4 ± 0.5 months, respectively. Percentage of T regulatory cells (Treg) and the expression of CD95 and CD25 in CD4^+^ lymphocytes were evaluated by flow cytometry. Lymphocyte proliferation capacity was measured by [^14^C]-thymidine incorporation and mRNA expression of IL-35, TGF-beta, IL-2 and IL-10 by real-time PCR. Lymphocyte proliferation was higher in OWC and OWE groups compared with the EC (3509 ± 887; 2694 ± 560, and 1768 ± 208 cpm, respectively) and EE (2313 ± 111 cpm) groups. CD95 expression on lymphocytes was augmented in the EC (953.9 ± 101.2) and EE groups (736.7 ± 194.6) compared with the OWC (522.1 ± 125.2) and OWE groups (551.6 ± 144.5). CTLA-4 expression was also lower in the OWC and OWE groups compared with the EC and EE groups. Percentage of Treg, IL-35, and IL-10 mRNA expression were lower in the OWC and OWE groups compared with the EC and EE groups. In conclusion, overweight children present altered immune system balance characterized by elevated lymphocyte proliferation due to a decrease in T regulatory cell percentage. These effects were partially reverted by moderate physical exercise, as demonstrated by decreased lymphocyte proliferation.

## Introduction

Obesity and physical inactivity are associated with an increased risk of development of several chronic diseases, such as *diabetes mellitus*, metabolic syndrome, cardiovascular diseases, and immune system disorders [1–6]. Sanchez et al. [7] described that adipose tissue has an important role in the production of mediators with inflammatory functions (adipokines), such as leptin, TNF-α and IL-6. Modifications of adipose tissue may alter leukocyte regulation leading to the development of inflammatory diseases [1, 8].

According to Marti et al. [6], obesity is related to alterations of the innate and acquired immune response, contributing to the deterioration of the immune system, in a similar manner to the immunosenescence process. In this case, pathological disorders such as autoimmune diseases can occur because of the lack of stimulus-induced T lymphocyte response or to the over-activation of these cells [9, 10]. Yudkin et al. [11] demonstrated that adipose tissue produced large amounts of IL-6 and this contributes to increased lymphocyte proliferation. Several T lymphocyte cell surface receptors are involved in cell activation control or apoptotic pathway induction. In fact, apoptosis inhibition in activated T lymphocytes may promote their accumulation, leading to exacerbated inflammation [12]. Studies have demonstrated an increase of peripheral activated T cells in obese mice [13, 14]. Probably these effects are related to the decreased regulatory T (Treg) cells in adipose tissue, as demonstrated by Deiullis et al. [15]. A failure in Treg cell (CD4^+^, CD25^+^, and Foxp3^+^) function can lead to an exacerbation of the immune response [16], promoting chronic inflammatory process state [17].

Physical exercise inhibits the immunosenescence process, and thereby reduces the incidence of several inflammatory diseases [7]. Studies have shown that regular moderate physical exercise modulates the number of circulating leukocytes in children and adults [8, 9]. In addition, Izadpanaha et al. [18] observed that a short-term physical exercise program intervention in children (age 13.0 ± 0.5 years) promotes a decrease in proinflammatory cytokines and metabolic risk factors, including IL-6, IL-8, TNFα, resistin, and leptin. However, there are no studies identifying possible effects of a moderate physical exercise program in lymphocyte subtypes of obese or overweight children.

It is well known that a sedentary lifestyle during childhood is a major problem related to the development of obesity. However, the differences on leukocyte function between healthy, overweight, and obese children that practice or not regularly physical activity have not been studied yet. Knowledge in this field will lead to a better understanding of the mechanisms invoved in the development of inflammatory disorders and will help to direct further studies aiming therapeutic interventions. In the present study, we compared the mechanisms of lymphocyte activation in overweight and eutrophic children involved with regular circus activities twice a week for a period of at least four months with children that are not involved with monitored physical exercises. Circus exercise is a leisure activity that could be an attractive form of intervention for children. These activities are characterized by artistic gymnastic movements. The main components include air acrobat movements, dance, climbing bar and equilibrium exercises, trapeze, and juggling with clubs and balls. Circus activities require both anaerobic and aerobic metabolism.

## Materials and Methods

### Subjects

Sixty female pubescent children, were chosen according to their stage of maturity [19, 20], classified by the self-evaluation as proposed by Matsudo et al [21]. All children of the present study were in Tanner stages II, III, or IV. Children were divided into four groups, accordingly to anthropometric measurements [22] and circus exercise practice in: Overweight Control (OWC), Eutrophic Control (EC), Overweight Exercise (OWE), and Eutrophic Exercise (EE). Volunteers with the following characteristics were excluded: autoimmune diseases, glucose intolerance, *diabetes mellitus*, or any other endocrine disease. Children from the exercised group practiced circus exercises twice a week for four to five months, in a Circus School, observing the frequency control. The OWE group practiced circus exercises for 4.3 ± 0.5 months and the EE group for 4.4 ± 0.5 months. There were no differences in relation to exercise period between OWE and EE. The intensity and the frequency of these activities were monitored during the last four months before blood sample collection in all groups of children. Children from the non-exercised groups did not participate in any extracurricular physical exercise program and were selected directly in the regular school.

The children’s parents signed the consent form regarding the procedures performed in this study. Experimental study procedure was performed in strict accordance with the Human Ethical Committee and was approved by the Ethical Committee of the Cruzeiro do Sul University (Certificate Number: 041/2010).

### Anthropometric parameters

Body mass index (BMI) was calculated from the weight and height measures [BMI = weight (kg]/height^2^ (m^2^)]. Skinfold measurements were taken at two sites: triceps (vertical fold midway between the olecranon and acromion processes) and subscapular (oblique skinfold, about one cm below the inferior angle of the scapula). Each skinfold measurement was repeated three times with Lange calipers (Lange Instrument Co., Inc., Lange, Cambridge USA). Circumferences were measured with an unstretchable metric tape. Waist circumference was measured at the narrowest part between the lower rib and the iliac crest (the natural waist). Abdominal circumference was measured at the level of the greatest anterior extension of the abdomen, which was usually at the level of the umbilicus.

To estimate and confirm the classification of groups, the percentage of body fat was determined using a tetrapolar bioimpedance (Biodynamics Corporation, 310, EUA). The bioimpedance measurements were performed as described by Lukaski [23]. The values are presented in Table 1.

**Table 1:** Anthropometric profile of eutrophic and overweight pubescent girls and intensity of the exercise.

PARAMETERS	EC	EE	OWC	OWE
	n = 15	n = 15	n = 15	n = 15
**Age (years)**	11.00 ± 0.29	10.60 ± 0.29	10.67 ± 0.22	10.00 ± 0.41
**Weight (kg)**	40.27 ± 1.63	40.79 ± 2.65	51.23 ± 2.03*	47.88 ± 2.70* #
**Height (m)**	1.51 ± 0.02	1.48 ± 0.03	1.49 ± 0.02	1.39 ± 0.03
**BMI (kg/m^2^)**	17.51 ± 0.47	16.80 ± 0.70	23.02 ± 0.68*	24.64 ± 0.88* #
**Body Fat (%)**	20.64 ± 1.60	11.91 ± 1.19*	29.89 ± 1.31*	31.10 ± 1.17* #
**Lean Mass (kg)**	32.94 ± 1.94	36.64 ± 2.64*	35.52 ± 1.60	33.58 ± 1.95
**Fat Mass (kg)**	9.22 ± 1.23	8.90 ± 0.53	15.33 ± 1.60*	14.67 ± 0.90* #
**Abdomen Circumference (cm)**	68.13 ± 1.61	59.94 ± 2.44*	80.20 ± 2.09*	72.28 ± 2.28* #
**Waist Circumference (cm)**	62.12 ± 1.10	58.35 ± 1.61	70.39 ± 1.36*	67.83 ± 1.26* #
**Tricep Skinfold (cm)**	15.08 ± 0.89	12.04 ± 1.06	24.17 ± 1.23*	22.36 ± 0.94* #
**Subscapular Skinfold (cm)**	10.31 ± 0.86	7.47 ± 0.83*	18.23 ± 1.10*	15.50 ± 0.73 * #
**Heart rate during exercise (bpm)**	---	173.93 ± 9.26	---	185.67 ± 6.91
**Heart rate reserve (bpm)**	---	122.73 ± 4.54	---	120.5 ± 4.88
**% of maximum heart rate reserve**	---	40.0 ± 4.2	---	52.0 ± 4.0 #

The values are presented as mean S.E.M.

*P< 0.01 vs. eutrophic.

^#^P < 0.05 vs. eutrophic exercise.

### Program of Regular Circus Physical Exercises

Training protocol consisted of regular circus physical exercises, during 60 min per session, twice a week. OWE group practiced circus exercises for 4.3 ± 0.5 months and the EE group for 4.4 ± 0.5 months. Attendance was controlled to ensure that children participated in the training for the last 4 months before blood sample collection and analysis. The sessions consisted of the following exercises: 10 min warm-up with recreational and folklore dance; 40 min with solo acrobatics, combination of jumps, spins, and twists on an acrobatic trampoline, balancing on a wooden leg, and air acrobatics on a trapeze, rope, and platform; and 10 min with stretching exercises.

To assess the intensity of the circus physical activities, each child was monitored twice a week with a heart rate (HR) monitor (Polar Vantage XL; Polar) during the last 16-week training period. The following values, in beats per minute (bpm), were determined: a) average resting HR (*HR rest*), measured during the initial rest interval of 60 seconds; b) average HR measured during the circus class (*HR during session*), c) average HR after one minute in the recovery period (*HR after 1 min*), measured one minute after the end of class, and d) variations of HR from one week to the next (*Δ = HR*), calculated for three situations (rest, during class, and after one min). After calculating the %HR reserve (i.e., the percentage of the difference between resting and maximal heart rate) [24], the training intensity was classified according to the American College of Sports Medicine (2000). Based on this calculation, intensity of circus physical exercises was classified as moderate. Results are presented in Table 1.

### Blood sample collection

Blood samples were collected from the exercised and non-exercised groups at least 48 hours after the last exercise bout. After overnight fasting (12 h), blood was collected from the antecubital vein into tubes containing ethylenediaminetetraacetic acid (EDTA, 1 mg/mL). Blood was centrifuged at 4°C, 400 *g*, for 10 min. Plasma samples were separated and frozen (−80°C) for further assays.

### Lymphocyte isolation

Lymphocytes were isolated from peripheral blood, as reported previously [25]. After plasma isolation, blood was diluted in phosphate-buffered saline (PBS, pH 7.4) (1:1), suspended in Histopaque-1077 (Sigma Chemical Co., St. Louis, MO, EUA), and centrifuged for 30 min, 400 *g*, at room temperature. Peripheral blood mononuclear cells (PBMC; a mixture of monocytes and lymphocytes) were collected from the interphase. Remanescent erytrocytes were lysed with 150 mM NH_4_CI, 10 mM NaHCO_3_, and 0.1 mM EDTA, at a pH of 7.4. PBMC were washed once with PBS and maintained in RPMI-1640 medium to allow adherence of the monocytes to the plates for further isolation of pure lymphocyte population from the supernatant (approximately 98% lymphocytes). The number of lymphocytes was determined using a Neubauer chamber under an optical microscope (Nikon, Melville, NY).

### Plasma glucose and insulin concentration

Plasma glucose was determined by colorimetric method using Glucose Assay Kit (Bioclin, Belo Horizonte, MG, BR—Catalog Number K082) as described by manufacturer. Insulin concentration was determined by Immunoradiometric assay (RIA) as described by the manufacturer protocol (Beckman Coulter, Catalog Number IM3210).

### Lymphocyte proliferation assay

Lymphocytes were maintained in RPMI-1640 enriched with 2 mM glutamine, buffered with 24 mM sodium bicarbonate, 20 mM HEPES, 10% fetal bovine serum (FBS), and antibiotics (10,000 U/mL penicillin and 10,000 μg/mL streptomycin). Cell culture was maintained at 37°C with 5% CO_2_ and 95% atmospheric air. Lymphocyte proliferation was determined by the incorporation of [2-^14^C]thymidine into the DNA. Lymphocytes were cultured in 96-well plates, 2.5 x 10^5^ cells per well. Cells were stimulated with 5 μg/mL concanavalin A (ConA), a classical T-lymphocyte mitogen [26]. The plates were incubated in a humidified atmospheric chamber with 5% CO_2_ and 95% air at 37°C. After 30 h [2-^14^C]-thymidine (1 μCi/mL) was added into medium, and the cells were further cultured for 18 h. After this period, lymphocytes were collected using the automatic cell collector Multiple Skatron Combi Cell Harvester (Suffolk, UK) and the incorporated radioactivity into DNA counted using tubes containing 1 mL of liquid scintillation in a Beckman LS-5000TD equipment (Beckman Instruments, Fullerton, CA, USA).

### Determination of cell membrane integrity

After isolation, lymphocytes (5 x 10^5^ cells) were centrifuged at 500 *g* for 15 min at 4°C and suspended in 500 μL PBS. Thereafter, 50 μL of a propidium iodide solution (50 mg/mL in PBS) was added, and the cells were analyzed using a FACSAria II flow cytometer (Becton Dickinson, San Juan, CA, USA). Ten thousand events were analyzed per experiment. Fluorescence from cells containing propidium iodide was evaluated using BD-Diva software (Becton Dickinson).

### DNA staining using propidium iodide

DNA fragmentation was analyzed by flow cytometry after DNA staining with propidium iodide according to Nicoletti et al. [27]. Cells were centrifuged at 400 *g*, for 15 min, at 4°C. Lymphocytes were gently suspended in 300 μL of hypotonic solution containing 50 mg/mL propidium iodide, 0.1% sodium citrate, and 0.1% Triton X-100. After that, cells were incubated for 2 h at 4°C. Fluorescence was then measured and analyzed as described above.

### CD95, CTLA-4, and T regulatory cell percentage determination

Lymphocytes were suspended in PBS and labeled with FITC-conjugated anti-CD95, APC-anti-CD25, or anti-CTLA-4 antibodies (1:50) (Becton Dickinson, San Juan, CA). Cells were incubated for 30 min at room temperature in the dark. Negative control cells were incubated with isotype-matched nonreactive IgG1 antibody. Thereafter, lymphocytes were washed with PBS and analyzed using a FACSAria II flow cytometer (Becton Dickinson, San Juan, CA). Ten thousands events were analyzed per experiment. Fluorescence from cells presenting FITC was evaluated using Diva software (Becton Dickinson).

The percentage of Treg cells was determined by the evaluation of CD4^+^, CD25^+^, and Foxp3^+^-positive cells. Intranuclear staining of Foxp3 was performed after fixation and permeabilization, according to the manufacturer’s protocol, and subsequently incubated with the specific antibody (Becton Dickinson).

### Determination of IL-35, IL-10, TGF-alpha, and IL-2 mRNA expression

Total RNA was obtained from lymphocytes (1x10^7^ cells per child) using 1 mL Trizol reagent (Invitrogen), as described by the manufacter’s protocol. After extraction, total RNA was treated with DNase AmpGrade enzyme for eliminating DNA contamination. The enzyme was added to 1 μg/μL into DNase buffer and the samples incubated for 30 min at 37°C. EDTA (25 mM) was added and incubated for 10 min at 65°C. Random Primer (0.2 pmoles/μL) was added to the RNA samples. Samples were incubated for 5 min at 65°C to prevent the formation of secondary and tertiary structures and to allow the annealing of primers to random samples. Afterwards, 10 mM dNTPs and 1 μL of the enzyme Revertaid MMuLV-RT (Fermentas) were added, resulting in a final reactive volume of 20 μL. The thermal cycler was programmed for three phases, keeping the first phase for 10 min at 25°C, the second phase for 60 min at 42°C for cDNA synthesis, and the third phase for 10 min at 70°C. At the end, samples were kept at 4°C into thermal cycler and transferred to the -80°C until further analysis.

mRNA expression was determined by PCR (Rotor Gene-3000; Corbett Research, Mortlake, Australia) using the Platinum SYBR Green qPCR SuperMix-UDG (Invitrogen). The sequences of the primers (Table 2) were designed using information from the GenBank public database of the National Center for Biotechnology Information. The relative quantification of each target gene was determined as previously described [28]. Beta2M gene was chosen as housekeeping gene in our study based on the analysis described in the GeNorm program, which determines the most stable reference gene from a set of potential genes in a given cDNA sample panel.

**Table 2:** The standardized conditions for RT-PCR analysis.

GeneBank accession number	Gene	Sense Primer	Antisense Primer	AT (°C)
NG012920	B2M	5'TGTCTGGGTTTCATCCATCCGACA	5'GCGGCATCTTCAAACCTCCATGA	63.9
NG012088	IL 10	5'GCTACGGCGCTGTCATCGATT	5'ATCCCAGAGCCCCAGATCCGAT	62.0
NC000003	IL 35	5'GTCTGCATCCAGCGGCTCGC	5'AGTTTGTCTGGCCTTCTGGAGCAT	62.5
NC007870	TGF	5'CCAGCGACTCGCCAGAGTGG	5'GGCCGGTAGTGAACCCGTTGA	62.5
M26062	IL 2	5’AATTACCTGGCACCACCTCGTC	5’TCCCTCCCTGAGTTCAAACGC	59.0

The sequences of the primers and the annealing temperature (AT) are shown for each gene under study.

### Analysis of plasma inflammatory markers

Plasma cytokine levels (TNF-α, IL-6, IL-2, IL-10, IL-4, and IL-8) were measured using ELISA kits (Quantikine, R&D System, Minneapolis, MN, USA). Limits of detection for the ELISA analyses were: 9.38 pg/mL for IL-6, 15.65 pg/mL for TNF-α, 31.2 pg/mL for IL-10, 15.6 pg/mL for IL-2, 31.2 pg/mL for IL-4, and 31.2 pg/mL for IL-8.

### Statistical analysis

Data are presented as the mean ± Standard Error of the Mean (SEM) and were analyzed by the Two-way analysis of variance (ANOVA) and the Bonferroni *post-hoc* test for parametric values. The Friedman test was used for non-parametric values. The values were calculated employing GraphPad Prism 5 software (Graph Pad Software, Inc., San Diego, CA, USA) and considered statistically significant when *P* < 0.05.

## Results

### Plasma glucose and insulin levels

There were no differences in plasma glucose and insulin concentration among groups. The glucose concentration was 75 ± 10; 83 ± 7; 78 ± 10; and 79 ± 8 mg/dL in EC, OWC, EE, and OWE groups, respectively. The insulin concentration was 16 ± 7; 20 ± 6; 18 ± 9 and 22 ±10 μUI/mL in EC, OWC, EE and OWE respectively. All values are in the normal range to the age.

### Lymphocyte viability

**Fig 1:**
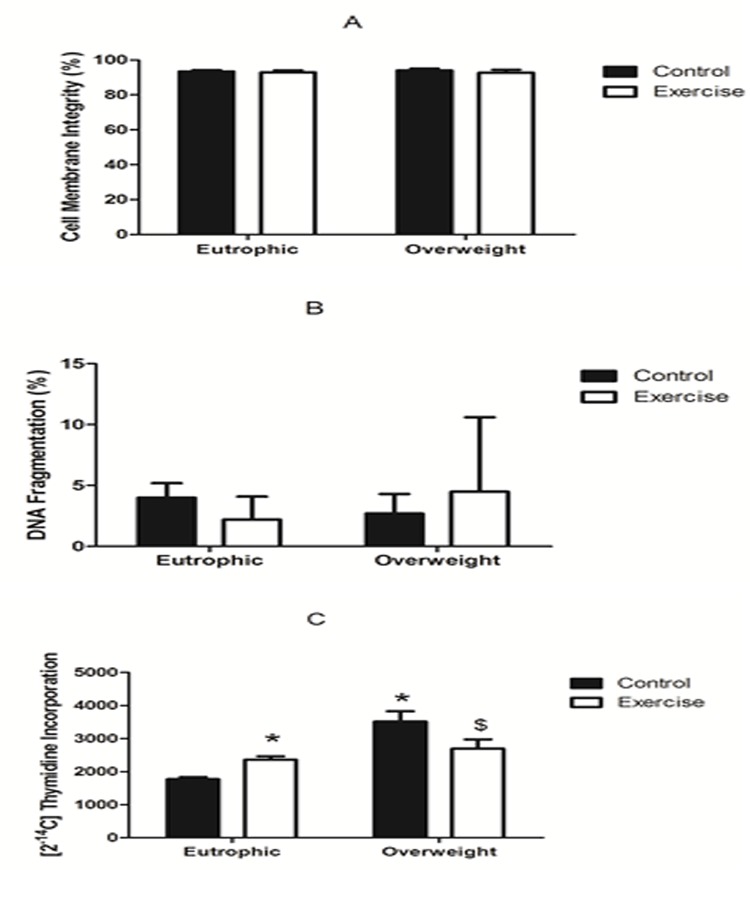
Lymphocyte viability and proliferation capacity in Overweight Control (OWC), Eutrophic Control (EC), Overweight Exercise (OWE), and Eutrophic Exercise (EE) children. (A) Membrane cell integrity. (B) DNA fragmentation. (C) Lymphocyte proliferation. Data are expressed as counts per minute (cpm). The values are presented as the mean ± S.E.M. *P < 0.01 vs. EC; ^$^P < 0.05 vs. OWC.

No difference in DNA fragmentation and membrane integrity of lymphocytes were found in lymphocytes from the four groups evaluated (Fig. 1A-B).

### Lymphocyte proliferative capacity

Proliferation of non-stimulated lymphocytes was not changed among the groups (data not shown). Higher proliferative response was observed in ConA-stimulated lymphocytes from OWC group as compared with the EC (59% higher) and OWE (20% higher) groups (Fig. 1C). Moreover, cell proliferation in the OWE group was not different from those from the EE and EC groups.

### Expression of CD95, CD25, and CTLA-4 in total lymphocytes

**Fig 2:**
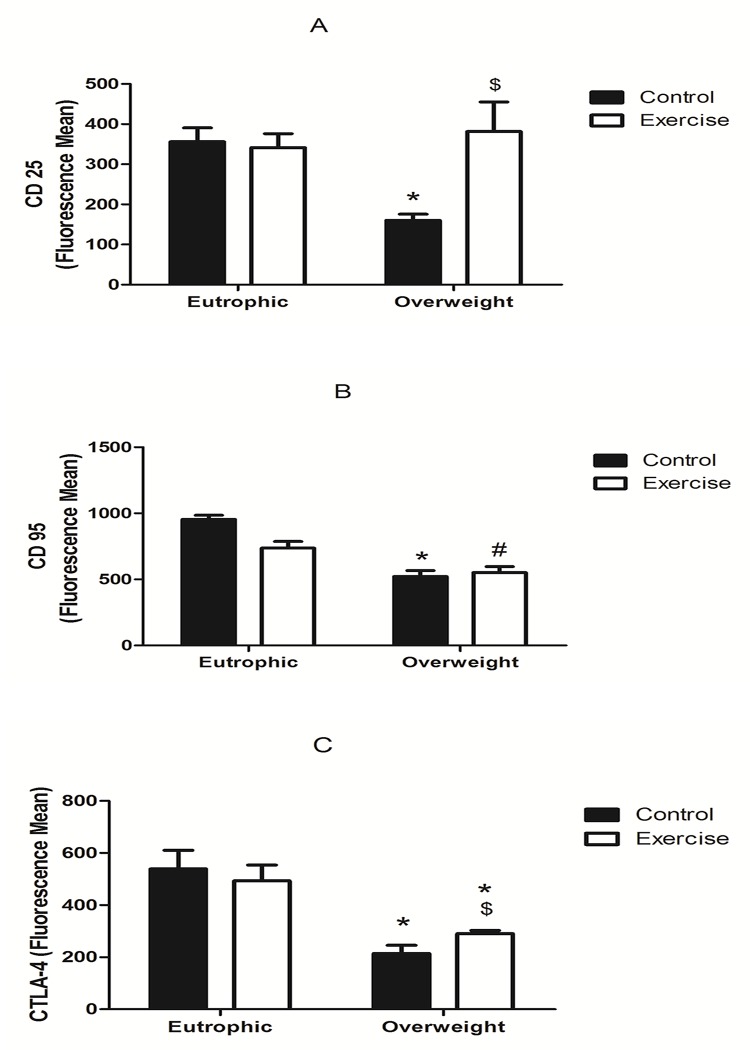
Expression of CD25 (A), CD95 (B), and CTLA-4 (C) in lymphocytes from Overweight Control (OWC), Eutrophic Control (EC), Overweight Exercised (OWE), and Eutrophic Exercised (EE) children. The values are presented as the mean ± S.E.M.; *P < 0.01 vs. EC; ^#^P < 0.05 vs. EE; ^$^P < 0.05 vs. OWC.

The OWC group presented a lower expression of CD25 in total peripheral lymphocytes when compared with the EC and EE groups (mean fluorescence values of 160 ± 16, 355.9 ± 34.7 and 341.1 ± 50.2 respectively). However, the OWE group (363.9 ± 151.4) showed a CD25 expression which was similar to the eutrophic groups and higher when compared with the OWC group (Fig. 2A).

CD95 expression in the EC (953.9 ± 101.2) and EE groups (736.7 ± 194.6) was higher as compared with the OWC (522. 1 ± 125.2) and OWE (551.6 ± 144.5) groups. There were no differences in expression of lymphocytes between exercised and non-exercised children in overweight and eutrophic groups (Fig. 2B).

CTLA-4 expression in the OWC and OWE groups (214.4 ± 31.3 and 304.4 ± 50.2, respectively) were lower than in the EC and EE groups (538.9 ± 63.8 and 492.7 ± 50.9, respectively). There were no differences in expression of lymphocytes between exercised and non-exercised children in overweight and eutrophic groups (Fig. 2C).

### Percentage of T regulatory lymphocytes

**Fig 3:**
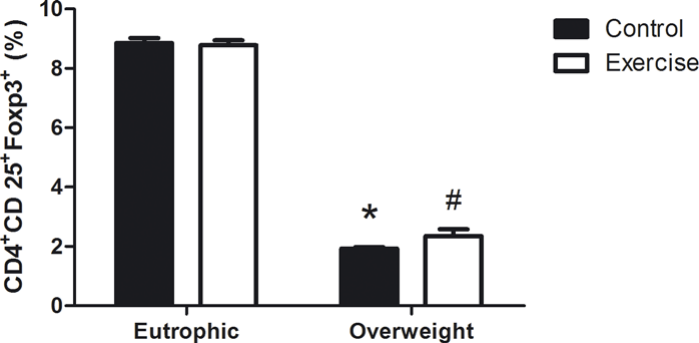
Percentage of T regulatory cells in Overweight Control (OWC), Eutrophic Control (EC), Overweight Exercised (OWE), and Eutrophic Exercised (EE) children. Cells were pelleted and labeled with FITC-conjugated anti-CD4, APC-conjugated anti-CD25, and PE-conjugated anti-Foxp3 and analyzed by flow cytometry. The values are presented as the mean ± S.E.M.; *P < 0.01 vs. EC; ^#^P < 0.05 vs. EE.

The percentages of CD4^+^, CD25^+^, and Foxp3^+^ T lymphocytes were evaluated. The OWC and OWE group presented a lower percentage of Treg cells (1.90 ± 0.05 and 2.35 ± 0.22) when compared with the EC and EE groups (8.9 ± 0.2 and 8.8 ± 0.2), as shown in Fig. 3. There were no differences in the expression of lymphocytes between EC and EE or OWC and OWE.

### Plasma TNF-α, IL-6, IL-2, IL-10, IL-4, and IL-8 levels

Plasma concentrations of TNF-α, IL-6, IL-2, IL-10, IL-4, and IL-8 were not different among the groups studied (Table 3).

**Table 3:** Plasma inflammatory markers in Overweight Control (OWC), Eutrophic Control (EC), Overweight Exercise (OWE), and Eutrophic Exercise (EE) children.

	EC		n	OWC		n	EE		n	OWE		n
TNF-α (pg/mL)	5.08	±17.74	15	11.32	±37.37	11	15.53	±44.91	15	26.44	±42.96	12
IL2 (pg/mL)	4.13	±9.73	15	6.96	±15.69	11	19.47	±52.73	15	15.63	±40.08	10
IL10 (pg/mL)	4.21	±3.06	15	3.63	±6.45	11	9.78	±17.53	15	5.32	±5.31	12
IL4 (pg/mL)	4.67	±1.60	15	4.14	±7.95	11	4,03	±3.03	10	2.73	±6.97	10
IL6 (pg/mL)	6.36	±3.61	15	1.69	±0.77	10	3.22	±1.98	10	4.12	±2.16	10
IL8 (pg/mL)	2.53	±1.58	15	5.17	±4.22	10	2.34	±1.48	10	3.01	±2.13	10

The values are presented as mean ± S.E.M..

n = number of children per group.

### IL-35, IL-2, IL-10, and TGF-beta mRNA expression in all lymphocytes

**Fig 4:**
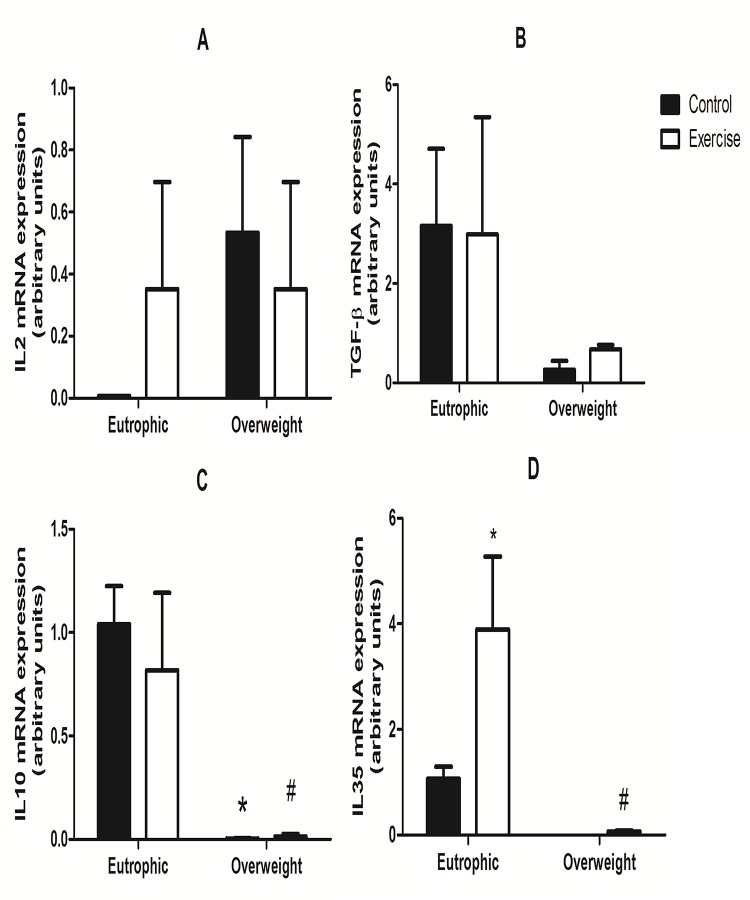
mRNA expression of IL-2 (A), TGF-beta (B), IL-10 (C), and IL-35 (D) in lymphocytes from Overweight Control (OWC), Eutrophic Control (EC), Overweight Exercised (OWE), and Eutrophic Exercised (EE) children. The analysis was performed by real-time RT-PCR. The values are presented as the mean ± S.E.M.;*P < 0.01 vs. EC; ^#^P < 0.05 vs. EE.

mRNA expression of important cytokines produced by Treg cells involved in lymphocyte differentiation was evaluated (Fig. 4). Production of IL-2 (Fig. 4A), TGF-beta (Fig. 4B), IL10 (Fig. 4C) and IL-35 (Fig. 4D) cytokines is related to Treg cell function. IL35 mRNA expression was 95% higher in the EE group when compared with the OWC and OWE groups. Moreover, IL35 mRNA expression of the EE group was 75% higher when compared with the EC group. IL10 mRNA levels were higher (95%) in the EC and EE groups when compared with the OWC and OWE groups (1.04 ± 0.18 and 0.82 ± 0.37, respectively). There were no differences between OWC and OWE in relation to all cytokine mRNA expression analyzed.

## Discussion

Obesity associated with a sedentary lifestyle can lead to changes in the immune system function exerted by T lymphocyte imbalance, favoring the development of chronic inflammatory diseases. The main finding observed in our study is that overweight children present a more pronounced lymphocyte activation related to a decrease in Treg cells and suppressor markers. On the other hand, these effects are partially resolved by regular physical activity, specifically circus activities, which attenuates the imbalance of immune system observed in overweight children.

Overweight children presented increased lymphocyte proliferative capacity when compared with eutrophic children. In the same way, Marti et al. [6] suggested that obesity is associated with abnormal immune function, as demonstrated by elevated leukocyte and T lymphocyte amount and reduced natural killer cell number, and cytotoxicity capacity. Herein, we showed that overweight exercised children present decreased proliferation capacity in stimulated cells when compared to overweight control children, suggesting that moderate physical exercise program may protect against exacerbated lymphocyte proliferation. Shimizu et al. [29] showed that exercise training at moderate intensity spontaneously increases Th CD28 expression, leading to the up-regulation of cytokine activity and Th cell proliferation and differentiation.

Whenever an increased lymphocyte proliferation is induced, an elevation in the suppressor T-lymphocyte cells (Treg cells) promotes the regulation of effector lymphocytes to guarantee the balance of immune function. We found that overweight children (OWC and OWE) have lower levels of Treg cells (CD4^+^, CD25^+^, and Foxp3^+^) than normal weight children. This effect is related to an imbalance of leukocyte control, since Treg cells are associated with the suppression of an exacerbated leukocyte response. Studies have demonstrated that the number of Treg cells is also decreased in adipose tissue from obese mice [30]. Our study is the first to evaluate the percentage and function of blood Treg cells in overweight children and the modulatory effect by a regular physical exercise program.

CD25 is the IL-2 receptor alpha chain expressed on activated T cells surface. Sakaguchi et al. [31] showed that T regulatory cells also express this receptor on its surface and participate to immune tolerance. In our study, we observed a decrease on CD25 expression on total peripheral lymphocytes in OWC. CD25 is undetectable in resting T-cells and its expression is stimulated by antigens or mitotic compounds, such as Concanavalin A [32]. However, when we evaluated the percentage of cells that express CD4^+^, CD25^+^ and Foxp3^+^ (Treg cells), we also observed a decrease, as described above. Probably, this decrease was due to the low level of Treg cells in blood circulation. In fact, studies have demonstrated that CD4^+^, CD25+ and Foxp3- cells do not have suppressive function and that these cells are related to a lymphocyte effector function [33].

Betini and Vignali [34] suggest that Treg cell activity is associated with TGF-beta, IL10, and IL35 production. We analyzed the mRNA expression of key cytokines released by Treg lymphocytes and observed that overweight children had low mRNA expression of IL35 and IL10 when compared with the eutrophic children. Mosser and Zhang [35] described the importance of IL10 produced by T cells in the inhibitory process of antigen-presenting cells. Moreover, IL10-knockout animals are more susceptible to the development of autoimmune diseases. Collison et al. [36] demonstrated that IL35 is related to the differentiation of T cells as well as to the inhibition of cell proliferation and Th1 and Th2 activation.

In addition, modulation of suppressive molecules may contribute to altered lymphocyte responses. CTLA-4 is also related to lymphocyte suppression and Treg cell differentiation. Karman et al. [37] showed that CTLA-4 negatively regulates T cell activation and that this protein also has an important role in Treg differentiation. In this study, a decreased expression of CTLA-4 in lymphocytes from overweight children was observed, according to the reduced Treg cells found in these children.

We also observed that CD95 expression in lymphocytes from overweight children was markedly lower when compared with eutrophic children. This receptor is related to the family of TNF-α receptors and is closely associated with the activation of cell death by apoptosis [38]. The activation of the CD95 pathways plays an important role in the regulation of lymphocyte activity, preventing excessive activation of these cells [39]. Our data indicate that, despite no alteration was observed in the cell death markers, reduced CD95 levels in overweight children can be related to exacerbated cell proliferation. Although a decrease in the proliferative capacity of lymphocytes from the exercised groups was observed, CD95 expression was not altered. Moreover, other studies [40] demonstrated that CD95 expression in lymphocytes is altered after a single session of moderate physical exercise (70% of VO_2_ max).

In order to determine the possible mediators involved with the altered lymphocyte activation in overweight children and the changes induced by circus physical exercise program, plasma concentrations of TNF-α, IL-2, IL-10, IL-8, and IL-6 were determined. Cytokine concentrations were not different among the groups. TNF-α levels are found to be elevated in obese adipose tissue, which probably occurs through a mechanism related to decreased adiponectin levels [41]. Adiponectin is secreted by adipose tissue and inhibits TNF-α expression in macrophages and adipocytes [42, 43]. Studies have shown this down-regulation on pro-inflammatory cytokine production [44] by adiponectin involves attenuation of NF-kB activation and subsequent translocation to the nucleus and inhibition of ERK1/2 activity as well [44]. Several studies showed that serum TNF-α levels are increased in obese individuals. However, most of these studies were conducted in animals or adult people [45, 46]. In contrast, our study demonstrated that the concentration of these cytokines in the plasma of overweight children is not different from that of the eutrophic children. Koistinen et al. [47] observed that TNF-α mRNA levels in subcutaneous adipose tissue is not different between lean and obese nondiabetic men. In agreement to our study, Breslin et al. [48] did not observe difference between eutrophic and overweight children. It is possible that the effects on plasma cytokines are more pronounced in obese than in eutrophic children, as shown by other authors [18, 49]. Interestingly, Russell et al. [50] observed that TNF-α receptors are elevated in obese adolescent girls, suggesting an easier way of TNF-α binding to its receptor and consequently a high effect without a significant increase in circulating TNF-α concentration, as also demonstrated by Hosick et al. [51]. In addition, cytokines perform autocrine and/or paracrine effects, which could help to explain the absence of significant difference in their concentration in plasma.

Although no differences on plasma cytokine concentrations were observed among the groups in our study, lymphocyte activity was altered. However, an imbalance on leukocyte function does not necessarily lead to the systemic inflammation. However, if the peripheral lymphocyte suppression mechanism fails, then the susceptibility for the development of inflammatory diseases increases. There are other plasma factors associated with the modulation of leukocyte function. Obesity-associated increase in the production of leptin (pro-inflammatory) and decrease in adiponectin (anti-inflammatory) modify the activation of immune cells [36]. Kraszula et al. [52] observed that patients with relapsing-remitting multiple sclerosis have high leptin and resistin levels, markedly low adiponectin levels, and consequently a reduced percentage/amount of Treg cells. Accordantly, increased plasma leptin levels have been observed by other authors in obese asthmatic, non asthmatic, and overweight children [53–55]].

In conclusion, overweight children present an altered immune system characterized by high lymphocyte proliferation due to a decrease in T regulatory cell amount. These effects are evidenced by reduced IL-10 levels and IL-35 mRNA expression and associated with decreased expression of suppressor proteins (CD95 and CTLA-4), leading to the inhibition of lymphocyte activation and differentiation. Overweight children submitted to regularly practice circus activities presented an attenuation of the immune system imbalance, by reducing lymphocyte proliferation activity and by preventing some deleterious effects in lymphocytes. Therefore, we conclude that overweight children are more prone to develop impaired immune system function, which is partially prevented by a regular and moderate physical exercise program.
